# Anticancer Drugs Approved by the US Food and Drug Administration From 2009 to 2020 According to Their Mechanism of Action

**DOI:** 10.1001/jamanetworkopen.2021.38793

**Published:** 2021-12-14

**Authors:** Timothée Olivier, Alyson Haslam, Vinay Prasad

**Affiliations:** 1 Department of Oncology, Geneva University Hospital, Geneva, Switzerland; 2 Department of Epidemiology and Biostatistics, University of California, San Francisco

## Abstract

**Importance:**

Both novel and next-in-class cancer drugs have a role in oncology, but the relative development of each is understudied.

**Objective:**

To characterize the mechanisms of action of anticancer drugs approved by the US Food and Drug Administration (FDA) between 2009 and 2020, noting how many approvals were based on a new mechanism of action vs next-in-class approvals.

**Design, Study, and Participants:**

This cross-sectional study included all anticancer drugs approved by the FDA from January 2009 to December 2020. The mechanism of action of each drug was extracted from FDA labels. Supportive-care treatments were excluded.

**Exposures:**

Name of drug approved, date of approval, indication, tumor type, mechanism of action, broad pharmaceutical class, and biological target. Approvals considering all tumor types and each tumor type separately were classified in 3 nonoverlapping categories: new mechanism of action, next in class, or subsequent approval.

**Main Outcomes and Measures:**

The number of all approvals each year; the number of approvals based on a new mechanism of action, either by drug (considering all tumor types) or by indication (considering tumor types separately); and the frequency of these numbers over time.

**Results:**

Overall, 332 approvals were included. Between 2009 and 2020, there was an increase in the total number of approvals from 8 to 57. We found that 209 approvals (63%) were for a next-in-class indication in a new tumor type (84 [25%]) or a subsequent indication of the same drug in the same tumor type (195 [59%]). When considering each tumor type separately, 123 approvals (37%) were based on a new mechanism of action.

**Conclusions and Relevance:**

In this study, approvals based on a new mechanism of action represented a minority of all approvals. Further consideration of incentives for drug development are needed to prioritize novel or highly innovative and transformative anticancer drugs.

## Key Points

**Question:**

How often are anticancer drugs approved based on a new mechanism of action?

**Findings:**

In this cross-sectional study of 332 US Food and Drug Administration–approved anticancer drugs from 2009 to 2020, 16% of approvals were based on a new mechanism of action when considering all tumor types and 37% when considering each tumor type separately. Overall, 63% of approvals were either a next-in-class drug within a tumor type or a subsequent indication of the same drug within a tumor type.

**Meaning:**

In this study, anticancer drugs using a new mechanism of action were a minority of all anti-cancer drugs approvals.

## Teaser

This cross-sectional study characterizes the mechanisms of action of anticancer drugs approved by the US Food and Drug Administration (FDA) between 2009 and 2020, noting how many approvals were based on a new mechanism of action vs next-in-class drugs.

## Introduction

The most impactful clinical improvements in anticancer drugs have occurred with the debut of treatments with a novel mechanism of action. New classes of cytotoxic chemotherapy (anthracyclines or taxanes) led to notable clinical improvements in many tumor types. Imatinib in chronic myeloid leukemia was the first and arguably remains the best, so far, kinase inhibitor.^1^ Monoclonal antibodies have provided major advances by targeting CD20 in B-lymphoma,^2^ *ERBB2*-positive breast cancer,^3,4^ and immune checkpoints.^5^

So-called next-in-class or me-too drugs define a new pharmaceutical compound with a known pharmaceutical class of treatment. Examples are numerous in medicine (eg, proton pump inhibitors, statins). Next-in class drug approvals raise several issues, such as the proportion of research and development that aids their efforts (in contrast to novel therapeutics), the role of next-in-class drugs in pricing, and questions about their safety and efficacy assessment vs the first-in-class compound.^6,7^

The US Food and Drug Administration (FDA) annual reports celebrate the increasing number of anticancer approvals.^8,9^ However, novel compounds are not distinguished from next-in-class drugs. In this article, we sought to systematically determine the mechanism of action for all anticancer drugs approved by the FDA from 2009 to 2020. We sought to describe the evolution of total number of approvals during this period. We specifically estimate the number of approvals based on a new mechanism of action vs next-in-class approvals or subsequent approvals of the same drug and their evolution over time.

## Methods

### Study Design and Research Strategy

This was a retrospective cross-sectional study of all anticancer drugs approved by the FDA from January 2009 through December 2020. The search was performed on June 9, 2021. We adhered to the Strengthening the Reporting of Observational studies in Epidemiology (STROBE) reporting guidelines. Because we used publicly available data and this was not human participant research, in accordance with 45 CFR §46.102(f), we did not submit this study to an institutional review board or require informed consent procedures.

### Identification and Selection of FDA Approvals

Approvals selected for the analysis needed to be an anticancer treatment, ie, drugs, including biologics. We excluded supportive-care treatment. We did include biosimilar approvals and approvals for other routes of administration for already approved drugs (eg, subcutaneous). The research was conducted using the FDA website and a previous systematic review.^10^ Data related to the drug, the cancer type, and the approval basis were extracted from FDA labels, review documents, package inserts, and when necessary, from PubMed.

### Mechanism of Action, Pharmaceutical Class, and Biological Target

The mechanism of action for each drug was examined using the FDA labels and review documents from the FDA website^19^and also from the World Health Organization anatomical therapeutic chemical–defined daily dose index 2021.^20^ Kinase inhibitors were classified either with a specific target (eg, anaplastic kinase lymphoma [ALK] inhibitor, RET inhibitor) or as pankinase inhibitor when a specific target could not be identified. The kinome represents all kinases that can be expressed in a single cell; there are more than 500 kinases in human cells.^11^ Kinase inhibitors have various molecular specificities for different kinase domains of proteins and can affect the kinome of a single patient differently.^12^ Kinase inhibitors may have several targets; this is so-called dirty targeted therapy.^13^ However, while no kinase inhibitor is exclusively specific to a single target, some compounds have been developed with a higher specificity.

To avoid misclassification errors or at least to mitigate bias by classifying all agents with the same method, we defined the following rules when attributing the mechanism of action of kinase inhibitors: (1) when multitargets were identified, with the development of the drug being made without a focus on a precise target, the kinase inhibitor was classified as a pankinase inhibitor and (2) when the drug was developed with a specific target, even if not 100% specific for this target, the drug was classified with this specific target (eg, RET inhibitors). After coding the mechanism of action, we classified every drug according to a general pharmaceutical class (eg, chemotherapy, monoclonal antibody, bispecific antibody, kinase inhibitor) and identified the biological target (eg, *ERBB2* was defined as the target for both monoclonal antibodies targeting *ERBB2* and kinase inhibitors targeting *ERBB2*).

### Classifications Based on Mechanism of Action

Each approval was classified according to the pharmaceutical mechanism of action in 3 nonoverlapping categories: (1) first approval of a compound with a novel mechanism of action; (2) first approval of a next-in-class compound; or (3) subsequent indication of an already approved compound (ie, within the study period or before the study period).

Based on these 3 nonoverlapping categories, we classified each approval with 2 methodological approaches. First, we classified approvals by drug, including all tumor types as a single category. For example, the approval of pembrolizumab for melanoma in 2014 made it the first anti–programmed cell death protein 1 (PD-1) monoclonal antibody to be approved across all tumor types. Thus, we classified it as a new mechanism of action approval. However, its approval for head and neck cancer in 2016, even though it was the first anti–PD-1 in this tumor type, was classified as a subsequent indication approval. Second, we classified approvals by indication; we classified each tumor type separately. Using the previous example, because pembrolizumab was the first anti–PD-1 monoclonal antibody to be approved in both melanoma (2014) and head and neck cancer (2016), we classified both approvals as a novel mechanism of action.

The first classification is based on the type of drug, regardless of the tumor type, while the second considers the indication of the approval in the first place and considers each tumor type separately. Consequently, some drugs could be coded as a new mechanism of action in the second approach and subsequent indication in the first approach. For the subsequent indication classification, we also reviewed whether the compound was approved before the study period and in which tumor type, using the same sources as for the identification and selection of FDA approvals.

### Data Abstraction

Information abstracted for each approval included the name of drug approved, date and year of approval, indication, tumor type, mechanism of action, broad pharmaceutical class, biological target, classification of approval considering all tumor types (ie, new mechanism of action, next in class, or subsequent approval), and classification of approval considering each tumor type separately (new mechanism of action, next in class, or subsequent approval). Two authors (T.O. and A.H.) made the selection and reviewed the FDA approvals for inclusion. One author (T.O.) abstracted the mechanism of action, pharmaceutical class, and biological target of each drug and conducted the 2 classifications of each approval. Any questions about the mechanism of action or the classification of approvals were discussed between all authors and were adjudicated, when necessary, by a third reviewer (V.P.).

### Statistical Analysis

The analysis was descriptive. Frequencies were calculated for categorical variables throughout. Most analyses were done using a calculator, with testing of differences in proportions between 2009 vs 2020 being done in R version 4.0.4 (R Project for Statistical Computing).

## Results

**Figure 1.**
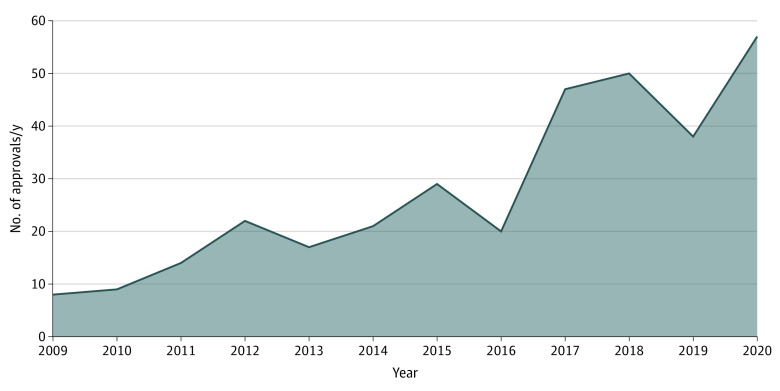
Total Number of Anticancer Drugs Approved by the US Food and Drug Administration From 2009 to 2020

There were 332 selected approvals. All selected approvals, with the drug name, date of approval, and indication, are available in the eAppendix in the Supplement. Across all anticancer drug approvals, we observed an increase in yearly cumulative number of FDA approved drugs between 2009 (8 approvals) and 2020 (57 approvals) (Figure 1).

**Figure 2.**
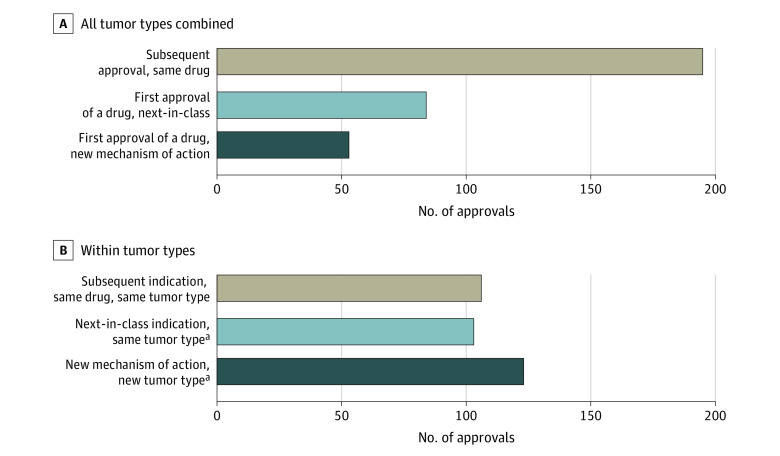
Mechanisms of Action for 332 Anticancer Drug Approvals Between 2009 and 2020

When examining individual drugs across tumor types, 195 approvals (59%) were subsequent approvals of the same drug, 84 (25%) were first approvals of a next-in-class drug, and 53 (16%) were approved based on a new mechanism of action (Figure 2A). While classifying approvals by indication (a drug can be novel in a different tumor type, if first approved for another), 106 (32%) approvals were subsequent approvals of the same drug in the same tumor type, 103 (31%) were first approvals within 1 tumor type of a next-in-class drug, while 123 (37%) drugs were approved based on a new mechanism of action in a new tumor type (Figure 2B).

**Figure 3.**
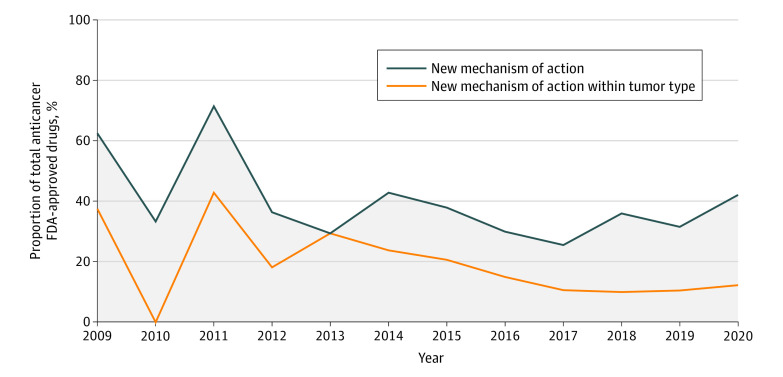
US Food and Drug Administration (FDA)–Approved Anticancer Drugs With New Mechanisms of Action From 2009 to 2020 A total of 3 anticancer drugs with a new mechanism of action were approved in 2009; 0 in 2010; 6 in 2011; 4 in 2012; 5 in 2013; 5 in 2014; 6 in 2015; 3 in 2016; 5 in 2017; 5 in 2018; 4 in 2019; and 7 in 2020. A total of 5 anticancer drugs with a new mechanism if action within a tumor type were approved in 2009; 3 in 2010; 10 in 2011; 8 in 2012; 5 in 2013; 9 in 2014; 11 in 2015; 6 in 2016; 12 in 2017; 18 in 2018; 12 in 2019; and 24 in 2020.

We then sought to estimate the cumulative number of approvals based on a new mechanism of action each year for all tumor types combined. In 2009, there were 3 approvals based on a new mechanism of action whatever the tumor type and 5 approvals based on a new mechanism of action in a new tumor type. In 2020, there were 7 approvals based on a new mechanism of action whatever the tumor type, and 24 approvals based on a new mechanism of action in a new tumor type (Figure 3).

We also sought to describe the proportion of approvals based on a new mechanism of action relative to all approvals (Figure 3). In 2009, 5 of 8 approvals (62.5%) were based on a new mechanism of action in a new tumor type, and 3 of 8 (37.5%) were based on a new mechanism of action whatever the tumor type; in 2020, they represented 24 of 57 (42.1%) and 7 of 57 (12.3%), respectively. The numerical difference in proportions between 2009 and 2020 did not met statistical significance in either case.

While classifying all 332 approvals based on their biological target, we identified 57 targets (eFigure 1 in the Supplement). Monoclonal antibodies targeting PD-1 or programmed cell death–ligand 1 (PD-L1) led to 65 approvals, representing 20% of all approvals. PD-1 represented the most frequent target of all approvals (49 approvals [15%]).

Additionally, among the 53 drugs approved based on a new mechanism of action, regardless of the tumor type, 50 distinct biological targets were identified, with 4 having already approved drugs before 2009 (*ERBB2*, CD20, the cell cycle for chemotherapy, and vascular endothelial growth factor [VEGF]). A total of 46 approvals were based on a new biological target during the study period.

We also examined approvals categorized based on their broad pharmaceutical class in eFigure 2 in the Supplement. Small-molecule inhibitors were the most approved drugs with 144 approvals (43%), 117 approvals (35%) were monoclonal antibodies, 23 (7%) were chemotherapeutic agents, 18 (5%) were antibody drug conjugates, and the remaining categories represented less than 5% each.

## Discussion

Our cross-sectional of first-in-class and next-in-class anticancer drugs approved by the FDA between 2009 and 2020 found a stepwise increase in the total number of approvals each year. This confirms a finding from a previous systematic review^10^ of FDA approvals until 2014.

We also found that despite increasing over time in absolute number, the proportion of approvals based on a novel mechanism of action decreased during the study period, when considering the novelty of the mechanism of action by drug for all tumor types combined, decreasing from 38% of all approvals in 2009 to 12% in 2020. When classifying less restrictively by indication (with each tumor type considered independently), the proportion of approvals based on a new mechanism of action also decreased with time from 62% in 2009 to 42% in 2020.

Even though there were 57 anticancer drug approvals in the year 2020 alone, our study identified only 46 approvals with a new biological target during a 12-year period. On average, the FDA is approving less than 4 new biological targets a year.

Next-in-class drugs may confer clinical efficacy in patients who are refractory to the first-in-class agents, may have different toxicity profiles compared with the first-in-class drugs, or offer new and more convenient routes of administration. However, issues regarding me-too or next-in-class drugs have been raised and are affecting medicine broadly.^6,7^ In our work, nearly one-third of all approvals (106 [32%]) were subsequent indications of the same drug for the same tumor type. In an analysis conducted by Hilal and colleagues,^14^ approvals were based on trials with an inappropriate use of crossover in 14% of them or an inappropriate control arm in 25% of them. Both limitations have the potential to alter conclusions about efficacy when a drug approved in one setting (eg, second line) is studied in another setting (eg, front line) or in combination with another drug. This raises concerns about the efficacy assessments for next-in-class approvals. When a me-too drug comes to market, by definition less postmarketing data are available than for the parent compound, providing less reliable data on safety than its already approved and prescribed first-in-class counterpart.

Global spending in anticancer drugs has increasing continuously, harming both the patient (financially and through reduced access and compliance) and society.^15^ Some have argued that brand-brand competition among drugs may eventually lead to lower costs. However, evidence has suggested, at least in the United States, that next-in-class drugs have no impact in lowering prices.^16^ Name-brand drugs are costlier than generic drug counterparts and are often heavily advertised.^17^ A study conducted by Mailankody and Prasad^18^ on the cost of anticancer FDA-approved drugs between 2009 and 2013 found no difference in median prices between next-in-class and novel mechanism of action drugs, suggesting that there may be underincentivization for the pharmaceutical industry to develop first-in-class compounds.^18^

Focusing on next-in-class drug development may result in persistent unmet medical needs.^7^ In our study, we found that 279 approvals (84%) were for a drug with a next-in-class or a subsequent indication. Moreover, while looking at each tumor type, we found that 209 approvals (63%) were a next-in-class indication or a subsequent indication of the same drug in the same tumor type, suggesting that most approvals compete with existing standard of care.^7,14^

### Limitations

This study has limitations. First, we restricted our analysis to 12-year period inclusion, which could be viewed as a limitation. However, previous studies have captured a wide range of anticancer drugs, including the period before the inclusion period of our study,^10^ and we sought to capture the most recent trend in anticancer treatment development. A second limitation is that we did not compare efficacy between first-in-class and next-in-class drugs or approvals. However, as many FDA approvals are not based on head-to-head comparison with the current standard of care,^14^ this research question was not studied because it was not central to our objectives. Third, the mechanism of action of some drugs may rely on some uncertainty, such as for kinase inhibitors. We tried to mitigate this bias by providing strict classification methods. Additionally, our analysis with all tumor types combined may have limitations regarding its clinical relevance. However, this analysis provides pharmacological and drug development perspectives that are valuable to our research question.

## Conclusions

In summary, this cross-sectional study of all anticancer drugs approved by the FDA between 2009 and 2020 found a stepwise increase in the total number of approvals. This contrasts with a decreasing proportion of approvals based on a novel mechanism of action, either when classifying the novelty of mechanism of action for all cancer or within each tumor type. Despite an increase in absolute number, approvals based on a new mechanism of action remain a minority of all approvals. Next-in-class drugs may provide benefit. They also have the potential to divert research and drug development from true innovation and to contribute to the increasing burden of cost of anticancer drugs. Furthermore, they often provide limited data about efficacy in comparison with their first-in-class compound, with shorter safety evaluations. Future research should consider incentives to encourage the pursuit of novel therapeutic targets.

## Notes

Supplement. **eAppendix.** Selected Approvals: Drug Name, Date of Approval, and Indication **eFigure 1.** Biological Targets for All Anticancer FDA Approvals Between 2009 and 2020 **eFigure 2.** Broad Pharmaceutical Class for All Anticancer FDA Approvals Between 2009 and 2020
