# Supplementary material for: Anticancer Drugs Approved by the US Food and Drug Administration From 2009 to 2020 According to Their Mechanism of Action
Source: JAMA Netw Open. 2021 Dec 14;4(12):e2138793. doi: 10.1001/jamanetworkopen.2021.38793 (PMC8672232; doi:10.1001/jamanetworkopen.2021.38793)
Supplement: Supplement. — eAppendix. Selected Approvals: Drug Name, Date of Approval, and Indication eFigure 1. Biological Targets for All Anticancer FDA Approvals Between 2009 and 2020 eFigure 2. Broad Pharmaceutical Class for All Anticancer FDA Approvals Between 2009 and 2020 [file jamanetwopen-e2138793-s001.pdf]

Supplemental Online Content

Olivier T, Haslam A, Prasad V. Anticancer drugs approved by the US Food and Drug Administration from 2009 to 2020 according to their mechanism of action. *JAMA Netw Open.* 2021;4(12):e2138793. doi:10.1001/jamanetworkopen.2021.38793

eAppendix. Selected Approvals: Drug Name, Date of Approval, and Indication

eFigure 1. Biological Targets for All Anticancer FDA Approvals Between 2009 and 2020

eFigure 2. Broad Pharmaceutical Class for All Anticancer FDA Approvals Between 2009 and 2020

This supplemental material has been provided by the authors to give readers additional information about their work.

© 2021 Olivier T et al. *JAMA Network Open.*

eAppendix. Selected Approvals: Drug Name, Date of Approval, and Indication

osimertinib 18.12.2020 adjuvant therapy after tumor resection in patients with non-small cell lung cancer (NSCLC) whose tumors have epidermal growth factor receptor (EGFR) exon 19 deletions or exon 21 L858R mutations
relugolix 18.12.2020 adult patients with advanced prostate cancer
selinexor 18.12.2020 in combination with bortezomib and dexamethasone for the treatment of adult patients with multiple myeloma who have received at least one prior therapy
margetuximab-cmkb 16.12.2020 metastatic HER2-positive breast cancer who have received two or more prior anti-HER2 regimens, at least one of which was for metastatic disease
pralsetinib 01.12.2020 adult and pediatric patients 12 years of age and older with advanced or metastatic RET-mutant medullary thyroid cancer (MTC) who require systemic therapy or RET fusion-positive thyroid cancer who require systemic therapy and who are radioactive iodine-refractory
naxitamab 25.11.2020 combination with granulocyte-macrophage colony-stimulating factor (GM-CSF) for pediatric patients one year of age and older and adult patients with relapsed or refractory high-risk neuroblastoma in the bone or bone marrow demonstrating a partial response, minor response, or stable disease to prior therapy
pembrolizumab 13.11.2020 in combination with chemotherapy for the treatment of patients with locally recurrent unresectable or metastatic triple-negative breast cancer (TNBC) whose tumors express PD-L1 (CPS ≥10)
venetoclax 16.10.2020 in combination with azacitidine, decitabine, or low-dose cytarabine (LDAC) for newly-diagnosed acute myeloid leukemia (AML) in adults 75 years or older, or who have comorbidities precluding intensive induction chemotherapy
pembrolizumab 14.10.2020 adult patients with relapsed or refractory classical Hodgkin lymphoma (cHL) and pediatric patients with refractory cHL, or cHL that has relapsed after 2 or more lines of therapy
nivolumab 02.10.2020 plus ipilimumab as first-line treatment for adult patients with unresectable malignant pleural mesothelioma
pralsetinib 04.09.2020 metastatic RET fusion-positive non-small cell lung cancer
azacitidine 01.09.2020 continued treatment of patients with acute myeloid leukemia who achieved first complete remission (CR) or complete remission with incomplete blood count recovery (CRi) following intensive induction chemotherapy and are not able to complete intensive curative therapy
carfilzomib and daratumumab 20.08.2020 in combination with dexamethasone for adult patients with relapsed or refractory multiple myeloma who have received one to three lines of therapy.
belantamab mafodotin-blmf 05.08.2020 adult patients with relapsed or refractory multiple myeloma who have received at least 4 prior therapies, including an anti-CD38 monoclonal antibody, a proteasome inhibitor, and an immunomodulatory agent
tafasitamab-cxix 31.07.2020 in combination with lenalidomide for adult patients with relapsed or refractory diffuse large B-cell lymphoma (DLBCL) not otherwise specified, including DLBCL arising from low grade lymphoma, and who are not eligible for autologous stem cell transplant
atezolizumab 30.07.2020 in combination with cobimetinib and vemurafenib for patients with BRAF V600 mutation-positive unresectable or metastatic melanoma
brexucabtagene autoleucel 24.07.2020 adult patients with relapsed or refractory mantle cell lymphoma
decitabine and cedazuridine 07.07.2020 myelodysplastic syndromes
avelumab 30.06.2020 maintenance treatment of patients with locally advanced or metastatic urothelial carcinoma (UC) that has not progressed with first-line platinum-containing chemotherapy
pembrolizumab 29.06.2020 first-line treatment of patients with unresectable or metastatic microsatellite instability-high (MSI-H) or mismatch repair deficient (dMMR) colorectal cancer
fixed-dose combination of pertuzumab, trastuzumab, and hyaluronidase–zzxf 29.06.2020 "Use in combination with chemotherapy as:

© 2021 Olivier T et al. *JAMA Network Open.*

neoadjuvant treatment of patients with HER2-positive, locally advanced, inflammatory, or early stage breast cancer (either greater than 2 cm in diameter or node positive) as part of a complete treatment regimen for early breast cancer;
adjuvant treatment of patients with HER2-positive early breast cancer at high risk of recurrence.
Use in combination with docetaxel for treatment of patients with HER2-positive metastatic breast cancer (MBC) who have not received prior anti-HER2 therapy or chemotherapy for metastatic disease."
pembrolizumab 24.06.2020 recurrent or metastatic cutaneous squamous cell carcinoma (cSCC) that is not curable by surgery or radiation
selinexor 22.06.2020 relapsed or refractory diffuse large B-cell lymphoma (DLBCL), not otherwise specified, including DLBCL arising from follicular lymphoma, after at least 2 lines of systemic therapy
tazemetostat 18.06.2020 adult patients with relapsed or refractory (R/R) follicular lymphoma (FL) whose tumors are positive for an EZH2 mutation as detected by an FDA-approved test and who have received at least 2 prior systemic therapies, and for adult patients with R/R FL who have no satisfactory alternative treatment options
pembrolizumab 16.06.2020 adult and pediatric patients with unresectable or metastatic tumor mutational burden-high (TMB H) [≥10 mutations/megabase (mut/Mb)] solid tumors, as determined by an FDA-approved test, that have progressed following prior treatment and who have no satisfactory alternative treatment options
gemtuzumab ozogamicin 16.06.2020 newly-diagnosed CD33-positive acute myeloid leukemia (AML) to include pediatric patients 1 month and older
lurbinectedin 15.06.2020 adult patients with metastatic small cell lung cancer (SCLC) with disease progression on or after platinum-based chemotherapy
nivolumab 10.06.2020 unresectable advanced, recurrent or metastatic esophageal squamous cell carcinoma (ESCC) after prior fluoropyrimidine- and platinum-based chemotherapy
ramucirumab 29.05.2020 in combination with erlotinib for first-line treatment of metastatic non-small cell lung cancer (NSCLC) with epidermal growth factor receptor (EGFR) exon 19 deletions or exon 21 (L858R) mutations
atezolizumab in combination with bevacizumab 29.05.2020 unresectable or metastatic hepatocellular carcinoma who have not received prior systemic therapy
nivolumab plus ipilimumab 26.05.2020 and 2 cycles of platinum-doublet chemotherapy as first-line treatment for patients with metastatic or recurrent non-small cell lung cancer (NSCLC), with no epidermal growth factor receptor (EGFR) or anaplastic lymphoma kinase (ALK) genomic tumor aberrations
brigatinib 22.05.2020 adult patients with anaplastic lymphoma kinase (ALK)-positive metastatic non-small cell lung cancer (NSCLC)
olaparib 19.05.2020 adult patients with deleterious or suspected deleterious germline or somatic homologous recombination repair (HRR) gene-mutated metastatic castration-resistant prostate cancer (mCRPC), who have progressed following prior treatment with enzalutamide or abiraterone
atezolizumab 18.05.2020 first-line treatment of adult patients with metastatic non-small cell lung cancer (NSCLC) whose tumors have high PD-L1 expression (PD-L1 stained ≥ 50% of tumor cells [TC ≥ 50%] or PD-L1 stained tumor-infiltrating immune cells [IC] covering ≥ 10% of the tumor area [IC ≥ 10%]), with no EGFR or ALK genomic tumor aberrations
ripretinib 15.05.2020 adult patients with advanced gastrointestinal stromal tumor (GIST) who have received prior treatment with 3 or more kinase inhibitors, including imatinib
rucaparib 15.05.2020 deleterious BRCA mutation (germline and/or somatic)-associated metastatic castration-resistant prostate cancer (mCRPC) who have been treated with androgen receptor-directed therapy and a taxane-based chemotherapy
nivolumab plus ipilimumab 15.05.2020 first-line treatment for patients with metastatic non-small cell lung cancer whose tumors express PD-L1(≥1%), as determined by an FDA-approved test, with no epidermal growth factor receptor (EGFR) or anaplastic lymphoma kinase (ALK) genomic tumor aberrations
pomalidomide 14.05.2020 adult patients with AIDS-related Kaposi sarcoma after failure of highly active antiretroviral therapy and Kaposi sarcoma in adult patients who are HIV-negative
olaparib 08.05.2020 first-line maintenance treatment of adult patients with advanced epithelial ovarian, fallopian tube, or primary peritoneal cancer who are in complete or partial response to first-line platinum-based chemotherapy and whose cancer is associated with homologous recombination deficiency positive status defined by either a deleterious or suspected deleterious BRCA mutation, and/or genomic instability
selpercatinib 08.05.2020 "Adult patients with metastatic RET fusion-positive non-small cell lung cancer (NSCLC);

© 2021 Olivier T et al. *JAMA Network Open.*

• Adult and pediatric patients ≥12 years of age with advanced or metastatic RET-mutant medullary thyroid cancer (MTC) who require systemic therapy;
• Adult and pediatric patients ≥12 years of age with advanced or metastatic RET fusion-positive thyroid cancer who require systemic therapy and who are radioactive iodine-refractory (if radioactive iodine is appropriate)"
capmatinib 06.05.2020 metastatic non-small cell lung cancer (NSCLC) whose tumors have a mutation that leads to mesenchymal-epithelial transition (MET) exon 14 skipping
daratumumab and hyaluronidase-fihj 01.05.2020 adult patients with newly diagnosed or relapsed/refractory multiple myeloma
niraparib 29.04.2020 maintenance treatment of adult patients with advanced epithelial ovarian, fallopian tube, or primary peritoneal cancer who are in a complete or partial response to first-line platinum-based chemotherapy
sacituzumab govitecan-hziy 22.04.2020 adult patients with metastatic triple-negative breast cancer who received at least two prior therapies for metastatic disease
ibrutinib 21.04.2020 combination with rituximab for the initial treatment of adult patients with chronic lymphocytic leukemia (CLL) or small lymphocytic lymphoma (SLL)
pemigatinib 20.04.2020 adults with previously treated, unresectable locally advanced or metastatic cholangiocarcinoma with a fibroblast growth factor receptor 2 (FGFR2) fusion or other rearrangement
tucatinib 17.04.2020 in combination with trastuzumab and capecitabine, for adult patients with advanced unresectable or metastatic HER2-positive breast cancer, including patients with brain metastases, who have received one or more prior anti-HER2-based regimens in the metastatic setting
mitomycin 15.04.2020 low-grade upper tract urothelial cancer
selumetinib 10.04.2020 pediatric patients, 2 years of age and older, with neurofibromatosis type 1 (NF1) who have symptomatic, inoperable plexiform neurofibromas
encorafenib 08.04.2020 in combination with cetuximab for the treatment of adult patients with metastatic colorectal cancer (CRC) with a BRAF V600E mutation
durvalumab 30.03.2020 in combination with etoposide and either carboplatin or cisplatin as first-line treatment of patients with extensive-stage small cell lung cancer
nivolumab and ipilimumab 10.03.2020 hepatocellular carcinoma (HCC) who have been previously treated with sorafenib
isatuximab-irfc 02.03.2020 in combination with pomalidomide and dexamethasone for adult patients with multiple myeloma who have received at least two prior therapies including lenalidomide and a proteasome inhibitor
neratinib 25.02.2020 in combination with capecitabine for adult patients with advanced or metastatic HER2-positive breast cancer who have received two or more prior anti-HER2 based regimens in the metastatic setting
tazemetostat 23.01.2020 adults and pediatric patients aged 16 years and older with metastatic or locally advanced epithelioid sarcoma not eligible for complete resection
avapritinib 09.01.2020 adults with unresectable or metastatic gastrointestinal stromal tumor (GIST) harboring a platelet-derived growth factor receptor alpha (PDGFRA) exon 18 mutation, including D842V mutations
pembrolizumab 08.01.2020 Bacillus Calmette-Guerin (BCG)-unresponsive, high-risk, non-muscle invasive bladder cancer (NMIBC) with carcinoma in situ (CIS) with or without papillary tumors who are ineligible for or have elected not to undergo cystectomy
olaparib 27.12.2019 maintenance treatment of adult patients with deleterious or suspected deleterious germline BRCA-mutated (gBRCAm) metastatic pancreatic adenocarcinoma, as detected by an FDA-approved test, whose disease has not progressed on at least 16 weeks of a first-line platinum-based chemotherapy regimen
fam-trastuzumab deruxtecan-nxki 20.12.2019 unresectable or metastatic HER2-positive breast cancer who have received two or more prior anti-HER2-based regimens in the metastatic setting
enfortumab vedotin-ejfv 18.12.2019 adult patients with locally advanced or metastatic urothelial cancer who have previously received a programmed death receptor-1 (PD-1) or programmed death-ligand 1 (PD-L1) inhibitor, and a platinum-containing chemotherapy in the neoadjuvant/adjuvant, locally advanced or metastatic setting
enzalutamide 16.12.2019 metastatic castration-sensitive prostate cancer

© 2021 Olivier T et al. *JAMA Network Open.*

atezolizumab 03.12.2019 in combination with paclitaxel protein-bound and carboplatin for the first-line treatment of adult patients with metastatic non-squamous non-small cell lung cancer (NSCLC) with no EGFR or ALK genomic tumor aberrations
acalabrutinib 21.11.2019 adults with chronic lymphocytic leukemia (CLL) or small lymphocytic lymphoma (SLL)
zanubrutinib 14.11.2019 adult patients with mantle cell lymphoma (MCL) who have received at least one prior therapy
niraparib 23.10.2019 advanced ovarian, fallopian tube, or primary peritoneal cancer treated with three or more prior chemotherapy regimens and whose cancer is associated with homologous recombination deficiency (HRD)-positive status
daratumumab 26.09.2019 adult patients with multiple myeloma in combination with bortezomib, thalidomide, and dexamethasone in newly diagnosed patients who are eligible for autologous stem cell transplant
apalutamide 17.09.2019 metastatic castration-sensitive prostate cancer
pembrolizumab plus lenvatinib 17.09.2019 advanced endometrial carcinoma that is not microsatellite instability high (MSI-H) or mismatch repair deficient (dMMR) and who have disease progression following prior systemic therapy but are not candidates for curative surgery or radiation
fedratinib 16.08.2019 adults with intermediate-2 or high-risk primary or secondary (post-polycythemia vera or post-essential thrombocythemia) myelofibrosis
entrectinib 15.08.2019 adults and pediatric patients 12 years of age and older with solid tumors that have a neurotrophic tyrosine receptor kinase (NTRK) gene fusion without a known acquired resistance mutation, are metastatic or where surgical resection is likely to result in severe morbidity, and have progressed following treatment or have no satisfactory standard therapy
pexidartinib 02.08.2019 tenosynovial giant cell tumor
pembrolizumab 30.07.2019 recurrent, locally advanced or metastatic, squamous cell carcinoma of the esophagus (ESCC) whose tumors express PD-L1 (Combined Positive Score [CPS] ≥10), as determined by an FDA-approved test, with disease progression after one or more prior lines of systemic therapy
darolutamide 30.07.2019 non-metastatic castration-resistant prostate cancer
selinexor 03.07.2019 in combination with dexamethasone for adult patients with relapsed or refractory multiple myeloma (RRMM) who have received at least four prior therapies and whose disease is refractory to at least two proteasome inhibitors, at least two immunomodulatory agents, and an anti-CD38 monoclonal antibody
daratumumab 27.06.2019 in combination with lenalidomide and dexamethasone for patients with newly diagnosed multiple myeloma who are ineligible for autologous stem cell transplant
pembrolizumab 17.06.2019 metastatic small cell lung cancer (SCLC) with disease progression on or after platinum-based chemotherapy and at least one other prior line of therapy
pembrolizumab 10.06.2019 first-line treatment of patients with metastatic or unresectable recurrent head and neck squamous cell carcinoma
polatuzumab vedotin-piiq 10.06.2019 in combination with bendamustine and a rituximab product for adult patients with relapsed or refractory diffuse large B-cell lymphoma (DLBCL), not otherwise specified, after at least two prior therapies
gilteritinib 29.05.2019 adult patients who have relapsed or refractory acute myeloid leukemia (AML) with a FLT3 mutation
lenalidomide 28.05.2019 in combination with a rituximab product for previously treated follicular lymphoma (FL) and previously treated marginal zone lymphoma
alpelisib 24.05.2019 in combination with fulvestrant for postmenopausal women, and men, with hormone receptor (HR)-positive, human epidermal growth factor receptor 2 (HER2)-negative, PIK3CA-mutated, advanced or metastatic breast cancer
venetoclax 15.05.2019 adult patients with chronic lymphocytic leukemia (CLL) or small lymphocytic lymphoma
avelumab 14.05.2019 in combination with axitinib for first-line treatment of patients with advanced renal cell carcinoma
ramucirumab 10.05.2019 single agent for hepatocellular carcinoma (HCC) in patients who have an alpha fetoprotein (AFP) of ≥ 400 ng/mL and have been previously treated with sorafenib
ado-trastuzumab emtansine 03.05.2019 adjuvant treatment of patients with HER2-positive early breast cancer (EBC) who have residual invasive disease after neoadjuvant taxane and trastuzumab-based treatment

© 2021 Olivier T et al. *JAMA Network Open.*

ivosidenib 02.05.2019 newly-diagnosed acute myeloid leukemia (AML) with a susceptible IDH1 mutation, as detected by an FDA-approved test, in patients who are at least 75 years old or who have comorbidities that preclude the use of intensive induction chemotherapy
pembrolizumab plus axitinib 19.04.2019 first-line treatment of patients with advanced renal cell carcinoma
erdafitinib 12.04.2019 locally advanced or metastatic urothelial carcinoma, with susceptible FGFR3 or FGFR2 genetic alterations, that has progressed during or following platinum-containing chemotherapy, including within 12 months of neoadjuvant or adjuvant platinum-containing chemotherapy
pembrolizumab 11.04.2019 first-line treatment of patients with stage III non-small cell lung cancer (NSCLC) who are not candidates for surgical resection or definitive chemoradiation or metastatic NSCLC. Patients' tumors must have no EGFR or ALK genomic aberrations and express PD-L1 (Tumor Proportion Score [TPS] ≥1%) determined by an FDA-approved test
atezolizumab 18.03.2019 in combination with carboplatin and etoposide, for the first-line treatment of adult patients with extensive-stage small cell lung cancer
atezolizumab 08.03.2019 PD-L1 positive unresectable locally advanced or metastatic triple-negative breast cancer
trastuzumab and hyaluronidase-oysk injection 28.02.2019 HER2 overexpressing breast cancer
trifluridine/ tipiracil tablets 22.02.2019 adult patients with metastatic gastric or gastroesophageal junction (GEJ) adenocarcinoma previously treated with at least two prior lines of chemotherapy that included a fluoropyrimidine, a platinum, either a taxane or irinotecan, and if appropriate, HER2/neu-targeted therapy
pembrolizumab 15.02.2019 adjuvant treatment of patients with melanoma with involvement of lymph node(s) following complete resection
cabozantinib 14.01.2019 hepatocellular carcinoma (HCC) who have been previously treated with sorafenib
tagraxofusp-erzs 21.12.2018 blastic plasmacytoid dendritic cell neoplasm (BPDCN) in adults and in pediatric patients 2 years and older.
calaspargase pegol-mknl 20.12.2018 component of a multi-agent chemotherapeutic regimen for acute lymphoblastic leukemia (ALL) in pediatric and young adult patients age 1 month to 21 years
olaparib 19.12.2018 maintenance treatment of adult patients with deleterious or suspected deleterious germline or somatic BRCA-mutated (gBRCAm or sBRCAm) advanced epithelial ovarian, fallopian tube or primary peritoneal cancer who are in complete or partial response to first-line platinum-based chemotherapy
pembrolizumab 19.12.2018 adult and pediatric patients with recurrent locally advanced or metastatic Merkel cell carcinoma
Herzuma 14.12.2018 biosimilar to Herceptin (trastuzumab, Genentech Inc.) for patients with HER2-overexpressing breast cancer
atezolizumab 06.12.2018 in combination with bevacizumab, paclitaxel, and carboplatin for the first-line treatment of patients with metastatic non-squamous, non-small cell lung cancer (NSq NSCLC) with no EGFR or ALK genomic tumor aberrations
gilteritinib 28.11.2018 adult patients who have relapsed or refractory acute myeloid leukemia (AML) with a FLT3 mutation
Truxima28.11.2018 biosimilar to Rituxan (rituximab, Genentech Inc.) for patients with CD20-positive, B-cell non-Hodgkin's lymphoma (NHL) to be used as a single agent or in combination with chemotherapy
larotrectinib 26.11.2018 adult and pediatric patients with solid tumors that have a neurotrophic receptor tyrosine kinase (NTRK) gene fusion without a known acquired resistance mutation, that are either metastatic
venetoclax 21.11.2018 in combination with azacitidine or decitabine or low-dose cytarabine for the treatment of newly-diagnosed acute myeloid leukemia (AML) in adults who are age 75 years or older, or who have comorbidities that preclude use of intensive induction chemotherapy
glasdegib 21.11.2018 in combination with low-dose cytarabine (LDAC), for newly-diagnosed acute myeloid leukemia (AML) in patients who are 75 years old or older or who have comorbidities that preclude intensive induction chemotherapy
brentuximab vedotin 16.11.2018 in combination with chemotherapy for previously untreated systemic anaplastic large cell lymphoma or other CD30-expressing peripheral T-cell lymphomas
pembrolizumab 09.11.2018 hepatocellular carcinoma (HCC) who have been previously treated with sorafenib
lorlatinib 02.11.2018 anaplastic lymphoma kinase (ALK)-positive metastatic non-small cell lung cancer (NSCLC) whose disease has progressed on crizotinib and at least one other ALK inhibitor for metastatic disease or whose disease has progressed on alectinib or ceritinib as the first ALK inhibitor therapy for metastatic disease

© 2021 Olivier T et al. *JAMA Network Open.*

pembrolizumab	30.10.2018	in combination with carboplatin and either paclitaxel or nab-paclitaxel as first-line treatment of metastatic squamous non-small cell lung cancer
talazoparib	16.10.2018	deleterious or suspected deleterious germline BRCA-mutated (gBRCAm), HER2 negative locally advanced or metastatic breast cancer
cemiplimab-rwlc	28.09.2018	metastatic cutaneous squamous cell carcinoma (CSCC) or locally advanced CSCC who are not candidates for curative surgery or curative radiation
dacomitinib tablets	27.09.2018	first-line treatment of patients with metastatic non-small cell lung cancer (NSCLC) with epidermal growth factor receptor (EGFR) exon 19 deletion or exon 21 L858R substitution mutations
duvelisib	24.09.2018	adult patients with relapsed or refractory chronic lymphocytic leukemia (CLL) or small lymphocytic lymphoma (SLL) after at least two prior therapies
duvelisib	24.09.2018	adult patients with relapsed or refractory follicular lymphoma (FL) after at least two prior systemic therapies
moxetumomab pasudotox-tdfk	13.09.2018	adult patients with relapsed or refractory hairy cell leukemia (HCL) who received at least two prior systemic therapies, including treatment with a purine nucleoside analog
pembrolizumab	20.08.2018	in combination with pemetrexed and platinum as first-line treatment of patients with metastatic, non-squamous non-small cell lung cancer (NSqNSCLC), with no EGFR or ALK genomic tumor aberrations
nivolumab	16.08.2018	metastatic small cell lung cancer (SCLC) with progression after platinum-based chemotherapy and at least one other line of therapy
lenvatinib capsules	16.08.2018	first-line treatment of patients with unresectable hepatocellular carcinoma
iobenguane I 131	30.07.2018	adult and pediatric patients (12 years and older) with iobenguane scan-positive, unresectable, locally advanced or metastatic pheochromocytoma or paraganglioma (PPGL) who require systemic anticancer therapy
ivosidenib	20.07.2018	adult patients with relapsed or refractory acute myeloid leukemia (AML) with a susceptible IDH1 mutation
ribociclib	18.07.2018	in combination with an aromatase inhibitor for pre/perimenopausal women with HR-positive, HER2-negative advanced or metastatic breast cancer, as initial endocrine-based therapy
enzalutamide	13.07.2018	castration-resistant prostate cancer
ipilimumab	10.07.2018	in combination with nivolumab for the treatment of patients 12 years of age and older with microsatellite instability-high (MSI-H) or mismatch repair deficient (dMMR) metastatic colorectal cancer (mCRC) that has progressed following treatment with a fluoropyrimidine, oxaliplatin, and irinotecan
encorafenib and binimetinib	27.06.2018	in combination for patients with unresectable or metastatic melanoma with a BRAF V600E or V600K mutation
pembrolizumab	13.06.2018	adult and pediatric patients with refractory primary mediastinal large B-cell lymphoma (PMBCL), or who have relapsed after two or more prior lines of therapy
bevacizumab	13.06.2018	epithelial ovarian, fallopian tube, or primary peritoneal cancer in combination with carboplatin and paclitaxel, followed by single-agent bevacizumab, for stage III or IV disease after initial surgical resection
pembrolizumab	12.06.2018	recurrent or metastatic cervical cancer with disease progression on or after chemotherapy whose tumors express PD-L1 (CPS ≥1)
venetoclax	08.06.2018	patients with chronic lymphocytic leukemia (CLL) or small lymphocytic lymphoma (SLL), with or without 17p deletion, who have received at least one prior therapy
dabrafenib plus trametinib	04.05.2018	anaplastic thyroid cancer with BRAF V600E mutation
tisagenlecleucel	01.05.2018	adult patients with relapsed or refractory large B-cell lymphoma after two or more lines of systemic therapy including diffuse large B-cell lymphoma (DLBCL) not otherwise specified, high grade B-cell lymphoma and DLBCL arising from follicular lymphoma
dabrafenib	30.04.2018	in combination for the adjuvant treatment of patients with melanoma with BRAF V600E or V600K mutations, as detected by an FDA-approved test, and involvement of lymph node(s), following complete resection
osimertinib	19.04.2018	first-line treatment of patients with metastatic non-small cell lung cancer (NSCLC) whose tumors have epidermal growth factor receptor (EGFR) exon 19 deletions or exon 21 L858R mutations

© 2021 Olivier T et al. *JAMA Network Open.*

nivolumab and ipilimumab 16.04.2018 intermediate or poor risk, previously untreated advanced renal cell carcinoma
rucaparib 06.04.2018 maintenance treatment of recurrent ovarian, fallopian tube, or primary peritoneal cancer
blinatumomab 29.03.2018 adult and pediatric patients with B-cell precursor acute lymphoblastic leukemia (ALL) in first or second complete remission with minimal residual disease (MRD) greater than or equal to 0.1%
nilotinib 22.03.2018 pediatric patients 1 year of age or older with newly diagnosed Philadelphia chromosome positive chronic myeloid leukemia in chronic phase (Ph+ CML-CP) or Ph+ CML-CP resistant or intolerant to prior tyrosine-kinase inhibitor (TKI) therapy
brentuximab vedotin 20.03.2018 adult patients with previously untreated stage III or IV classical Hodgkin lymphoma (cHL)
abemaciclib 26.02.2018 in combination with an aromatase inhibitor as initial endocrine-based therapy for postmenopausal women with hormone receptor (HR)-positive, human epidermal growth factor receptor 2 (HER2)-negative advanced or metastatic breast cancer
durvalumab 16.02.2018 unresectable stage III non-small cell lung cancer (NSCLC) whose disease has not progressed following concurrent platinum-based chemotherapy and radiation therapy
apalutamide 14.02.2018 non-metastatic castration-resistant prostate cancer
abiraterone acetate 07.02.2018 in combination with prednisone for metastatic high-risk castration-sensitive prostate cancer
Lu 177 dotatate 26.01.2018 somatostatin receptor-positive gastroenteropancreatic neuroendocrine tumors (GEP-NETs), including foregut, midgut, and hindgut neuroendocrine tumors in adults
afatinib 12.01.2018 broadened indication in first-line treatment of patients with metastatic non-small cell lung cancer (NSCLC) whose tumors have non-resistant epidermal growth factor receptor (EGFR) mutations
olaparib tablets 12.01.2018 deleterious or suspected deleterious germline BRCA-mutated (gBRCAm), HER2-negative metastatic breast cancer who have been treated with chemotherapy either in the neoadjuvant, adjuvant, or metastatic setting
pertuzumab 20.12.2017 in combination with trastuzumab and chemotherapy as adjuvant treatment of patients with HER2-positive early breast cancer at high risk of recurrence
nivolumab 20.12.2017 adjuvant treatment of patients with melanoma with involvement of lymph nodes or in patients with metastatic disease who have undergone complete resection
bosutinib 19.12.2017 patients with newly-diagnosed chronic phase (CP) Philadelphia chromosome positive (Ph+) chronic myelogenous leukemia (CML)
cabozantinib 19.12.2017 advanced renal cell carcinoma
Ogivri 01.12.2017 biosimilar to Herceptin (trastuzumab, Genentech, Inc.) for the treatment of patients with HER2-overexpressing breast or metastatic stomach cancer (gastric or gastroesophageal junction adenocarcinoma)
sunitinib malate 16.11.2017 adjuvant treatment of adult patients at high risk of recurrent renal cell carcinoma following nephrectomy
obinutuzumab 16.11.2017 in combination with chemotherapy, followed by obinutuzumab monotherapy in patients achieving at least a partial remission, for the treatment of adult patients with previously untreated stage II bulky, III, or IV follicular lymphoma
dasatinib 09.11.2017 pediatric patients with Philadelphia chromosome-positive (Ph+) chronic myeloid leukemia (CML) in the chronic phase
brentuximab vedotin 09.11.2017 adult patients with primary cutaneous anaplastic large cell lymphoma (pcALCL) or CD30-expressing mycosis fungoides (MF) who have received prior systemic therapy
alectinib 06.11.2017 patients with anaplastic lymphoma kinase (ALK)-positive metastatic non-small cell lung cancer
acalabrutinib 31.10.2017 adult patients with mantle cell lymphoma (MCL) who have received at least one prior therapy
axicabtagene ciloleucel 18.10.2017 adult patients with relapsed or refractory large B-cell lymphoma after two or more lines of systemic therapy, including diffuse large B-cell lymphoma (DLBCL) not otherwise specified, primary mediastinal large B-cell lymphoma, high-grade B-cell lymphoma, and DLBCL arising from follicular lymphoma

© 2021 Olivier T et al. *JAMA Network Open.*

abemaciclib 28.09.2017 in combination with fulvestrant for women with HR-positive, HER2-negative advanced or metastatic breast cancer with disease progression following endocrine therapy
nivolumab 22.09.2017 hepatocellular carcinoma
pembrolizumab 22.09.2017 recurrent locally advanced or metastatic, gastric or gastroesophageal junction adenocarcinoma whose tumors express PD-L1
cabazitaxel 14.09.2017 in combination with prednisone for the treatment of patients with metastatic castration-resistant prostate cancer previously treated with a docetaxel-containing treatment regimen
copanlisib 14.09.2017 adult patients with relapsed follicular lymphoma who have received at least two prior systemic therapies
Mvasi 14.09.2017 biosimilar to Avastin
gemtuzumab ozogamicin 01.09.2017 relapsed or refractory CD33-positive AML in adults and in pediatric patients 2 years and older
gemtuzumab ozogamicin 01.09.2017 newly-diagnosed CD33-positive acute myeloid leukemia (AML) in adults
tisagenlecleucel 30.08.2017 patients up to age 25 years with B-cell precursor acute lymphoblastic leukemia (ALL) that is refractory or in second or later relapse
olaparib tablets 17.08.2017 maintenance treatment of adult patients with recurrent epithelial ovarian, fallopian tube, or primary peritoneal cancer, who are in a complete or partial response to platinum-based chemotherapy
inotuzumab ozogamicin 17.08.2017 adults with relapsed or refractory B-cell precursor acute lymphoblastic leukemia
liposome-encapsulated combination of daunorubicin and cytarabine 03.08.2017 adults with newly-diagnosed therapy-related AML (t-AML) or AML with myelodysplasia-related changes (AML-MRC), two types of AML having a poor prognosis
enasidenib 01.08.2017 adult patients with relapsed or refractory acute myeloid leukemia with an isocitrate dehydrogenase-2 (IDH2) mutation
nivolumab 01.08.2017 patients 12 years and older with mismatch repair deficient (dMMR) and microsatellite instability high (MSI-H) metastatic colorectal cancer that has progressed following treatment with a fluoropyrimidine, oxaliplatin, and irinotecan
neratinib 17.07.2017 extended adjuvant treatment of adult patients with early stage HER2-overexpressed/amplified breast cancer, to follow adjuvant trastuzumab-based therapy
blinatumomab 11.07.2017 relapsed or refractory B-cell precursor acute lymphoblastic leukemia (ALL) in adults and children
dabrafenib and trametinib 22.06.2017 metastatic non-small cell lung cancer (NSCLC) with BRAF V600E mutation
rituximab and hyaluronidase human 22.06.2017 adult patients with follicular lymphoma, diffuse large B-cell lymphoma, and chronic lymphocytic leukemia
ceritinib 26.05.2017 metastatic non-small cell lung cancer (NSCLC) whose tumors are anaplastic lymphoma kinase (ALK)-positive
pembrolizumab 23.05.2017 adult and pediatric patients with unresectable or metastatic, microsatellite instability-high (MSI-H) or mismatch repair deficient (dMMR) solid tumors that have progressed following prior treatment and who have no satisfactory alternative treatment options or with MSI-H or dMMR colorectal cancer that has progressed following treatment with a fluoropyrimidine, oxaliplatin, and irinotecan
pembrolizumab 18.05.2017 locally advanced or metastatic urothelial carcinoma who have disease progression during or following platinum-containing chemotherapy or within 12 months of neoadjuvant or adjuvant treatment with platinum-containing chemotherapy
pembrolizumab 10.05.2017 in combination with pemetrexed and carboplatin for the treatment of patients with previously untreated metastatic non-squamous non-small cell lung cancer
avelumab 09.05.2017 locally advanced or metastatic urothelial carcinoma whose disease progressed during or following platinum-containing chemotherapy or within 12 months of neoadjuvant or adjuvant platinum-containing chemotherapy
durvalumab 01.05.2017 locally advanced or metastatic urothelial carcinoma who have disease progression during or following platinum-containing chemotherapy or who have disease progression within 12 months of neoadjuvant or adjuvant treatment with platinum-containing chemotherapy
midostaurin 28.04.2017 adult patients with newly diagnosed acute myeloid leukemia (AML) who have a specific genetic mutation called FLT3, in combination with chemotherapy

© 2021 Olivier T et al. *JAMA Network Open.*

brigatinib 28.04.2017 anaplastic lymphoma kinase (ALK)-positive metastatic non-small cell lung cancer (NSCLC) who have progressed on or are intolerant to crizotinib
regorafenib 27.04.2017 Expanded indication to include the treatment of patients with hepatocellular carcinoma (HCC) who have been previously treated with sorafenib
palbociclib 31.03.2017 hormone receptor (HR) positive, human epidermal growth factor receptor 2 (HER2) negative advanced or metastatic breast cancer in combination with an aromatase inhibitor as initial endocrine-based therapy in postmenopausal women
osimertinib 30.03.2017 metastatic epidermal growth factor receptor (EGFR) T790M mutation-positive non-small cell lung cancer (NSCLC), as detected by an FDA-approved test, whose disease has progressed on or after EGFR tyrosine kinase inhibitor (TKI) therapy
niraparib 27.03.2017 maintenance treatment (intended to delay cancer growth) of adult patients with recurrent epithelial ovarian, fallopian tube or primary peritoneal cancer, whose tumors have completely or partially shrunk (complete or partial response, respectively) in response to platinum-based chemotherapy
avelumab 23.03.2017 adults and pediatric patients 12 years and older with metastatic Merkel cell carcinoma
pembrolizumab 15.03.2017 adult and pediatric patients with refractory classical Hodgkin lymphoma (cHL), or those who have relapsed after three or more prior lines of therapy
ribociclib 13.03.2017 in combination with an aromatase inhibitor as initial endocrine-based therapy indicated for the treatment of postmenopausal women with hormone receptor (HR)-positive, human epidermal growth factor receptor 2 (HER2)-negative advanced or metastatic breast cancer
lenalidomide 22.02.2017 maintenance therapy for patients with multiple myeloma following autologous stem cell transplant
nivolumab 02.02.2017 locally advanced or metastatic urothelial carcinoma who have disease progression during or following platinum-containing chemotherapy or have disease progression within 12 months of neoadjuvant or adjuvant treatment with platinum-containing chemotherapy
rucaparib 19.12.2016 deleterious BRCA mutation (germline and/or somatic)–associated advanced ovarian cancer who have been treated with two or more chemotherapies
daratumumab 21.11.2016 in combination with lenalidomide (Revlimid) and dexamethasone, or bortezomib (Velcade) and dexamethasone, for the treatment of patients with multiple myeloma who have received at least one prior therapy
nivolumab 10.11.2016 patients with recurrent or metastatic squamous cell carcinoma of the head and neck with disease progression on or after a platinum-based therapy
pembrolizumab 24.10.2016 metastatic non–small cell lung cancer whose tumors express programmed cell death ligand 1 (PD-L1)
olaratumab 19.10.2016 soft-tissue sarcoma not amenable to curative treatment with radiotherapy or surgery and with a histologic subtype for which an anthracycline-containing regimen is appropriate
atezolizumab 18.10.2016 metastatic non–small cell lung cancer whose disease has progressed during or following platinum-containing chemotherapy. Patients with epidermal growth factor receptor (EGFR) or anaplastic lymphoma kinase (ALK) genomic tumor aberrations should have disease progression on an FDA-approved therapy for these aberrations prior to receiving atezolizumab
erlotinib 18.10.2016 non–small cell lung cancer, in particular, to limit its use to patients whose tumors have specific EGFR mutations
nivolumab 13.09.2016 dosage for the currently approved indications in renal cell carcinoma, metastatic melanoma, and non–small cell lung cancer modified to 240 mg intravenously every 2 weeks
pembrolizumab 05.08.2016 recurrent or metastatic head and neck squamous cell carcinoma with disease progression on or after platinum-containing chemotherapy
atezolizumab 18.05.2016 locally advanced or metastatic urothelial carcinoma who have disease progression during or following platinum-containing chemotherapy or have disease progression within 12 months of neoadjuvant or adjuvant treatment with platinum-containing chemotherapy
nivolumab 17.05.2016 classical Hodgkin lymphoma that has relapsed or progressed after autologous hematopoietic stem cell transplantation and posttransplantation brentuximab vedotin (Adcetris)

© 2021 Olivier T et al. *JAMA Network Open.*

lenvatinib 13.05.2016 in combination with everolimus (Afinitor), for the treatment of patients with advanced renal cell carcinoma following one prior antiangiogenic therapy

cabozantinib 25.04.2016 advanced renal cell carcinoma in patients who have received prior antiangiogenic therapy

venetoclax 11.04.2016 patients with chronic lymphocytic leukemia with 17p deletion, as detected by an FDA-approved test, who have received at least one prior therapy

crizotinib 11.03.2016 metastatic non–small cell lung cancer whose tumors are ROS1-positive

everolimus 26.02.2016 adult patients with progressive, well-differentiated, nonfunctional neuroendocrine tumors of gastrointestinal or lung origin with unresectable, locally advanced, or metastatic disease

obinutuzumab 26.02.2016 in combination with bendamustine followed by obinutuzumab monotherapy for the treatment of patients with follicular lymphoma who have relapsed after, or are refractory to, a rituximab (Rituxan)-containing regimen

palbociclib 19.02.2016 in combination with fulvestrant (Faslodex) for the treatment of women with hormone receptor–positive, HER2-negative advanced or metastatic breast cancer with disease progression following endocrine therapy

eribulin 28.01.2016 unresectable or metastatic liposarcoma who have received a prior anthracycline-containing regimen

ofatumumab 19.01.2016 extended treatment of patients who are in complete or partial response after at least two lines of therapy for recurrent or progressive chronic lymphocytic leukemia

pembrolizumab 18.12.2015 first-line treatment of patients with unresectable or metastatic melanoma

alectinib 01.12.2015 NSCLC

nivolumab 23.11.2015 single agent, of patients with BRAF V600 wild-type unresectable or metastatic melanoma

cobimetinib 01.11.2015 in combination with the BRAF inhibitor vemurafenib (Zelboraf) to treat metastatic or unresectable melanoma in patients whose tumors express the BRAF V600E or V600K mutatio

daratumumab 01.11.2015 duplicate from above

elotuzumab 01.11.2015 in combination with two other therapies to treat patients with multiple myeloma who have received one to three prior medications

ixazomib 01.11.2015 in combination with lenalidomide and dexamethasone for the treatment of patients with multiple myeloma who have received at least one prior therapy

nivolumab 01.11.2015 metastatic renal cell carcinoma whose disease progressed on an antiangiogenic therapy

necitumumab 01.11.2015 in combination with gemcitabine and cisplatin, for first-line treatment of patients with metastatic squamous non-small cell lung cancer

osimertinib 01.11.2015 metastatic epidermal growth factor receptor (EGFR) T790M mutation-positive non-small cell lung cancer (NSCLC)

ipilimumab 28.10.2015 cutaneous melanoma with pathologic involvement of regional lymph nodes of more than 1 mm who have undergone complete resection

talimogene laherparepvec 01.10.2015 unresectable cutaneous, subcutaneous, and nodal lesions in patients with melanoma recurrent after initial surgery

pembrolizumab 01.10.2015 NSCLC

irinotecan liposome injection 01.10.2015 in combination with fluorouracil and leucovorin to treat patients with metastatic pancreatic cancer whose disease has progressed after gemcitabine-based chemotherapy

trabectedin 01.10.2015 liposarcoma or leiomyosarcoma

nivolumab/ipilimumab 30.09.2015 BRAF V600 wild-type, unresectable or metastatic melanoma

trifluridine and tipiracil 01.09.2015 metastatic colorectal cancer who have been previously treated with fluoropyrimidine-, oxaliplatin- and irinotecan-based chemotherapy, an anti-VEGF biological therapy, and if RAS wild-type, an anti-EGFR therapy

brentuximab vedotin 17.08.2015 post–autologous hematopoietic stem cell transplantation consolidation treatment for patients with classical Hodgkin lymphoma at high risk of relapse or progression

© 2021 Olivier T et al. *JAMA Network Open.*

gefitinib	13.07.2015	first-line treatment of patients with metastatic non-small cell lung cancer (NSCLC) whose tumours harbor specific types of epidermal growth factor receptor (EGFR) gene mutations
sonidegib	01.07.2015	locally advanced basal cell carcinoma (BCC) not amenable to curative surgery or radiation therapy
ramucirumab	24.04.2015	in combination with FOLFIRI for metastatic CRC whose disease has progressed on a first line bevacizumab, oxaliplatin, and fluoropyrimidine containing regimen
nivolumab	01.03.2015	advanced (metastatic) non-small-cell lung cancer (NSCLC) whose disease progressed during or after platinum-based chemotherapy
dinutuximab	01.03.2015	pediatric patients with high-risk neuroblastoma who achieve at least a partial response to prior first-line multiagent, multimodality therapy
daratumumab	01.02.2015	multiple myeloma
panobinostat	01.02.2015	multiple myeloma
panobinostat	01.02.2015	in combination with bortezomib (Velcade) and dexamethasone for the treatment of patients with multiple myeloma who have received at least two prior standard therapies
palbociclib	01.02.2015	in combination with letrozole, as initial endocrine-based therapy for postmenopausal women with estrogen receptor (ER)-positive, human epidermal growth factor receptor 2 (HER2)-negative metastatic breast cancer
lenvatinib	01.02.2015	locally recurrent or metastatic, progressive, radioactive iodine-refractory (RAI-refractory) differentiated thyroid cancer
ibrutinib	01.01.2015	Waldenström's macroglobulinemia
lanreotide	16.12.2014	unresectable well or moderately differentiated locally advanced or metastatic GEP-NETs (gastroenteropancreatic neuroendocrine tumors)
ramucirumab	12.12.2014	in combination with docetaxel for metastatic NSCLC with disaese progression on or after platinum based chemo
ruxolitinib	04.12.2014	polycythemia vera
blinatumomab	01.12.2014	Philadelphia chromosome-negative relapsed /refractory B cell precursor acute lymphoblastic leukemia
olaparib	01.12.2014	ovarian
nivolumab	01.12.2014	melanoma
ramucirumab	05.11.2014	in combination with paclitaxel for advanced gastric or gastroesophageal junction adenocarcinoma
bevacizumab	01.11.2014	in combination with paclitaxel, pegylated liposomal doxorubicin, or topotecan for the treatment of patients with platinum-resistant, recurrent epithelial ovarian, fallopian tube, or primary peritoneal cancer
pembrolizumab	01.09.2014	unresectable or metastatic melanoma with disease progression following treatment with ipilimumab (Yervoy) and, in BRAF V600 mutation–positive patients after treatment with a BRAF inhibitor
bevacizumab	01.08.2014	persistent, recurrent, or metastatic cervical cancer in combination with paclitaxel and cisplatin or paclitaxel and topotecan
idelalisib	23.07.2014	relapsed CLL in combiation with rituximab, for whom rituximab alone would be onsidered appropriate therapy due to other comorbidities
belinostat	01.07.2014	relapsed or refractory peripheral T-cell lymphoma (PTCL)
idelalisib	01.07.2014	relapsed follicular B-cell non-Hodgkin lymphoma or relapsed small lymphocytic lymphoma (SLL) who have received at least two prior systemic therapies
idelalisib	01.07.2014	traditional approval to treat patients with relapsed chronic lymphocytic leukemia (CLL). Used in combination with rituximab (Rituxan); accelerated approval to treat patients with relapsed follicular B-cell non-Hodgkin lymphoma and relapsed small lymphocytic lymphoma. Idelalisib is intended to be used in patients who have received at least two prior systemic therapies
mercaptopurine	28.04.2014	ALL
ramucirumab	21.04.2014	monotherapy for previously treated patients with advanced or metastatic gastric or gastroesophageal junction adenocarcinoma
ofatumumab	17.04.2014	previously untreated patients with CLL for whom fludarabine based therapy is considered inappropriate
ramucirumab	01.04.2014	advanced or metastatic gastric cancer or gastroesophageal junction adenocarcinoma with disease progression on or after prior treatment with fluoropyrimidine- or platinum-containing chemotherapy

© 2021 Olivier T et al. *JAMA Network Open.*

ceritinib 01.04.2014 NSCLC
ibrutinib 01.02.2014 CLL
trametinib 10.01.2014 in combination in the treatment of unresectable or metastatic melanoma with BRAF V600 E or K mutation
sorafenib 22.11.2013 locally recurrent or metastatic, progressive, differentiated thyroid carcinoma (DTC) refractory to radioactive iodine treatment
crizotinib 20.11.2013 ALK-positive advanced NSCLC who had previously received one platinum-containing regimen
ibrutinib 13.11.2013 mantle cell lymphoma (MCL) who have received at least one prior therapy
obinutuzumab 01.11.2013 in combination with chlorambucil for the treatment of patients with previously untreated chronic lymphocytic leukemia
pertuzumab 30.09.2013 in combination with trastuzumab (Herceptin) and docetaxel for patients with early-stage breast cancer in the neoadjuvant setting
nab-paclitaxel 06.09.2013 in combination with gemcitabine for the first line treatment of patients with metastatic adenocarcinoma of the pancreas
afatinib 12.07.2013 first-line treatment of patients with metastatic non-small cell lung cancer (NSCLC) whose tumors have epidermal growth factor receptor (EGFR) exon 19 deletions or exon 21 (L858R) substitution mutations
lenalidomide 05.06.2013 mantle cell lymphoma (MCL) who have relapsed or whose disease has progressed after two prior therapies including at least one prior treatment with bortezomib
dabrafenib 29.05.2013 melanoma whose tumors express the BRAF V600E gene mutation
trametinib 29.05.2013 melanoma with BRAF V600E or V600K gene mutations, single agent
radium 2232 dichloride 15.05.2013 castration resistant prostate cancer, symptomatic bone metastases and no known visceral metastatic disease
erlotinib 14.05.2013 first line treatment of metastatic NSCLC whose tumors have EGFR exon 19/21 substitution mutations
regorafenib 25.02.2013 Locally advanced, unresectable or metastatic gastrointestinal stromal tumor (GIST) who have been previously treated with imatinib mesylate and sunitinib malate
ado-trastuzumab emtansine 22.02.2013 single agent for the treatment of HER2 positive metastatic breast cancer who previously received trastuzumab and a taxane (separately or in combination)
pomalidomide 08.02.2013 multiple myeloma whose disease progressed after being treated with other cancer drugs, who have received at least two prior therapies, including lenalidomide (Revlimid) and bortezomib (Velcade), and whose disease did not respond to treatment and progressed within 60 days of the last treatment (relapsed and refractory)
doxorubicin hydrochloride 04.02.2013 ovarian cancer in patients whose disease has progressesd or recurred after platinum chemo or intolerance
bevacizumab 23.01.2013 in combination with fluoropyrimidine-irinotecan or fluoropyrimidine-oxaliplatin based chemo for the treatment of patients with metastatic CRC whose disease has progressed on a first-line bevacizumab containing regimen
ponatinib 17.12.2012 CP-CML that is resistant or intolerant to prior tyrosine kinase inhibitor therapy of PH+ ALL that is resistant or intolerant to prior tyrosine kinase inhibitor therapy
ponatinib 14.12.2012 T315I-positive chronic-phase, accelerated-phase, or blast-phase chronic myeloid leukemia (CML) or T315I-positive Philadelphia chromosome–positive acute lymphoblastic leukemia (ALL), as well as patients with chronic-phase, accelerated-phase, or blast-phase CML or Philadelphia chromosome–positive ALL
abiraterone acetate 10.12.2012 in combination with predinisone for the treatment of patients with metastatic castration resistant prostate cancer
cabozantinib 29.11.2012 progressive metastatic medullary thyroid cancer
rituximab 19.10.2012 NHL who did not experience a grade 3 or 4 infusion-related adverse reaction during cycle 1
pemetrexed 17.10.2012 locally advanced or metastatic NSCLC followed by pemetrexed maintenance in patients whose disease has not progressed after 4 cycles of platinum and pemetrexed as first-line chemo
nab-paclitaxel 11.10.2012 in combination with carboplatin for the initial treatment of patients with locally advanced or metastatic non–small cell lung cancer (NSCLC) who are not candidates for curative surgery or radiation therapy

© 2021 Olivier T et al. *JAMA Network Open.*

regorafenib 27.09.2012 metastatic CRC who have been previously treated with fluoropyrimidine, oxaliplatin, and irinotecan based chemo, and anti-BEGF therapy, and if KRAS wild type, and anti-EGFR therapy
bosutinib 04.09.2012 adult patients with chronic, accelerated, or blast phase Ph+ chronic myelogenous leukemia (CML) with resistance or intolerance to prior therapy
enzalutamide 31.08.2012 metastatic castration resistant prostate cancer who have previously received docetaxel
everolimus 30.08.2012 tuberous sclerosis complex who have subependymal giant cell astrocytoma
liposomal vincristine 09.08.2012 adult patients with Philadelphia chromosome-negative (Ph-) acute lymphoblastic leukemia (ALL) in second or greater relapse or whose disease has progressed following two or more anti-leukemia therapie
ziv-aflibercept injection 03.08.2012 in combination with 5-fluorouracil, leucovorin, irinotecan, for the treatment of patients with metastatic CRC that is resitant to or has progressed following an oxaliplatin containing regimen
everolimus 20.07.2012 in combination with exemestane to treat postmenopausal women with advanced hormone receptor–positive, HER2-negative breast cancer after failure of treatment with letrozole or anastrozole
carfilzomib 20.07.2012 multiple myeloma who have received at least two prior therapies including bortezomib (Velcade) and an immunomodulatory agent and exhibited disease progression during or within 60 days of completing their last therapy
cetuximab 01.07.2012 in combination with FOLFIRI for first line treatment of patients with K-ras mutation negative (wild-type), EGFR expressing metastatic CRC
pertuzumab 08.06.2012 in combination with trastuzumab (Herceptin, Genentech) and docetaxel for the treatment of patients with HER2-positive metastatic breast cancer (MBC) who have not received prior anti-HER2 therapy or chemotherapy for metastatic disease
pazopanib 26.04.2012 advanced soft-tissue sarcoma who have previously received chemotherapy
everolimus 26.04.2012 TS renal angiomyolipoma
imatinib mesylate 31.01.2012 adjuvant treatment of adults following complete resection of Kit positive GIST
vismodegib 30.01.2012 adults with mBCC (basal cell carcinoma) or with laBCC with recurrence following surgery or who are not candidates for surgery or radiation
axitinib 27.01.2012 advanced renal cell carcinoma [RCC] after failure of one prior systemic therapy
asparaginase Erwina chrysanthemi 18.11.2011 component of a multi-agent chemo regimen for the treatment of patients with ALL who have developed hypersensitivity to E. coli-derived asparaginase
ruxolitinib 16.11.2011 intermediate and high risk myelofibrosis
cetuximab 07.11.2011 in combination with platinum-based therpy plus 5-florouracil for the first-line treatment of patients with recurrent locoregional disease and/or metastatic squamous cell carcinoma of the head and neck
crizotinib 26.08.2011 locally advanced or metastatic non-small cell lung cancer (NSCLC) that is anaplastic lymphoma kinase (ALK)-positive as detected by an FDA-approved test
brentuximab vedotin 19.08.2011 relapsed or refractory Hodgkin lymphoma
brentuximab vedotin 19.08.2011 systemic anaplastic large cell lymphoma
vemurafenib 17.08.2011 unresectable or metastatic melanoma with the BRAF V600E mutation
sunitinib20.05.2011 progressive, well-differentiated pancreatic neuroendocrine tumors (pNETs) in patients with unresectable locally advanced or metastatic disease
everolimus 05.05.2011 progressive pancreatic neuroendocrine tumors that cannot be removed by surgery or that have metastasized
abiraterone acetate 28.04.2011 in combination with predinsone for the treatment of patients with metastatic castration resistant prostate cancer who have received prior chemo containing docetaxel
vandetanib 06.04.2011 medullary thyroid cancer that cannot be removed by surgery or that has spread to other parts of the body

© 2021 Olivier T et al. *JAMA Network Open.*

peginterferon alfa-2b	29.03.2011	adjuvant treatment of melanoma with microscopic or gross nodal involvement within 84 days of definitive surgical resection including complete lymphadenectomy
ipilimumab	25.03.2011	unresctable or metastatic melanoma
rituximab	28.01.2011	maintenance therapy for patients with previously untreated follicular CD-20 positive, B-cell non-Hodkin lymphoma who achieve a response to rituximab in combination with chemotherapy
eribulin mesylate	15.11.2010	metastatic breast cancer who have previously received an antracycline and a taxane in either the adjuvant or metastatic setting and at least 2 chemo regimens for the treatment of metastatic disease
everolimus	29.10.2010	pediatric dosage form of everolimus (Afinitor), is used to treat patients with subependymal giant cell astrocytoma
dasatinib	28.10.2010	newly diagnosed adult patients with PH+ CML in chronic phase
trastuzumab	20.10.2010	combination with cisplatin and a lflouropyrimidine for the treatment of patients with HER2 overexpressing metastatic gastric or gastroesophageal junction adenocarcinoma, who have not received prior treatment for metastatic disease
nilotinib	17.06.2010	newly dianosed PH+ CML in chronic phase
cabazitaxel	17.06.2010	in combination with predisone to treat metastaic hormone-refractory prostate cancer previously treated with a docetaxel cocntaining regimen
erlotinib	16.04.2010	maintenance treatment of patients with locally advanced or metastatic NSCLC whose disease has not progressed after 4 cycles of platinum-based first-line chemo
rituximab	18.02.2010	in combination with fludarabine and cyclophosphamide for the treatment of patients with CLL
lapatinib	29.01.2010	in combination with letrozole for the treatment of postmenopausal women with hormone receptor positive metastatic breast cancer that overexpresses the HER2 receptor for whom hormonal therapy is indicated
romidepsin	06.11.2009	cutaneous T-cell lymphoma (CLL)
ofatumumab	26.10.2009	CLL refractory to fludarabine and alemtuzumab
pazopanib	19.10.2009	advanced RCC
pralatrexate	24.09.2009	relapsed or refractory peripheral T-cell lymphoma (PTCL)
bevacizumab	31.07.2009	in combination with interferon alfa for the treatment of patients with metastatic RCC
pemetrexed	02.07.2009	maintenance treatment of locally advanced or metastatic NSCLC paitnest with no disease progression after 4 cycles of platinum-based first line chemotherapy
bevacizumab	05.05.2009	glioblastoma with progressive disease following prior therapy
everolimus	31.03.2009	RCC after failure of treatment with sunitinib or sorafenib

© 2021 Olivier T et al. *JAMA Network Open.*

eFigure 1: Biological Targets for All Anticancer FDA Approvals Between 2009 and 2020

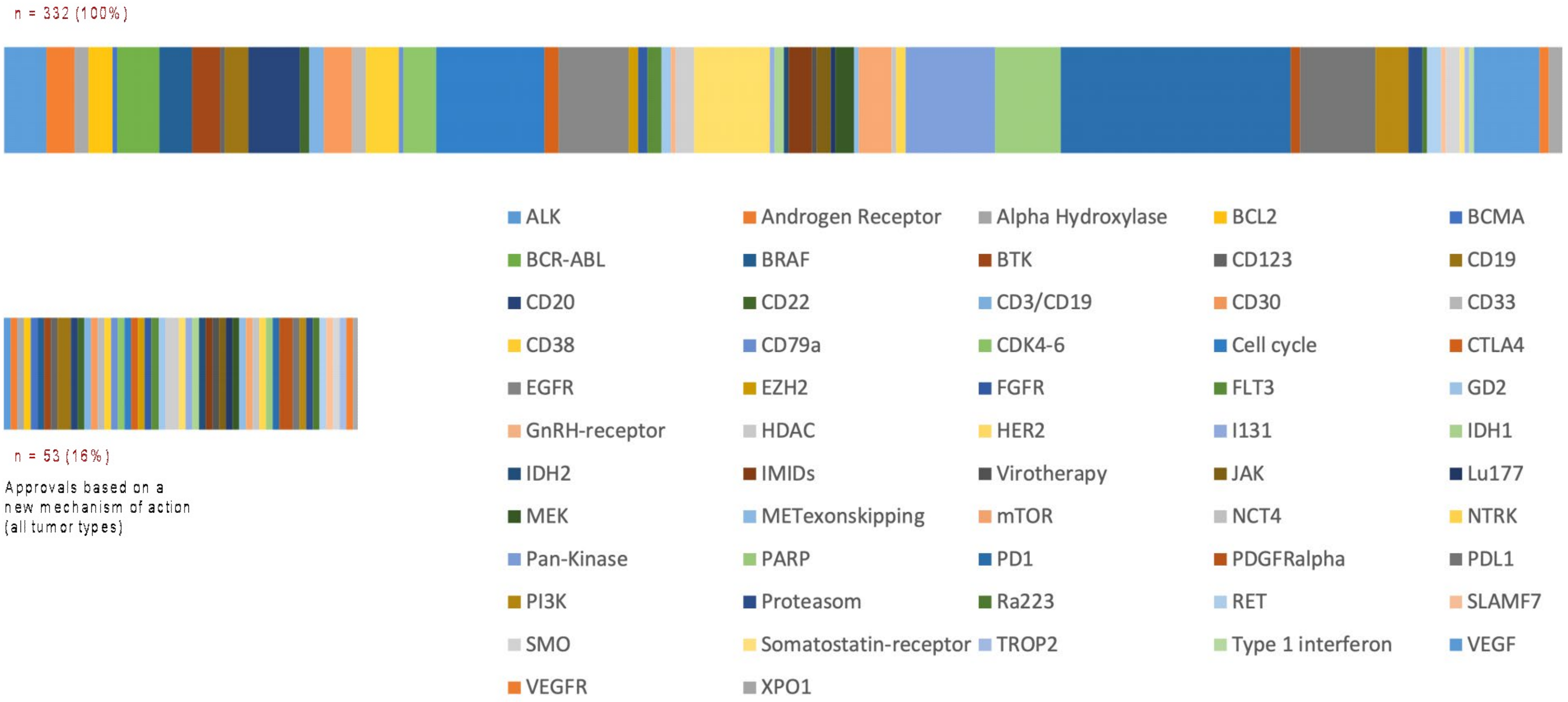

© 2021 Olivier T et al. *JAMA Network Open.*

eFigure 2. Broad Pharmaceutical Class for All Anticancer FDA Approvals Between 2009 and 2020

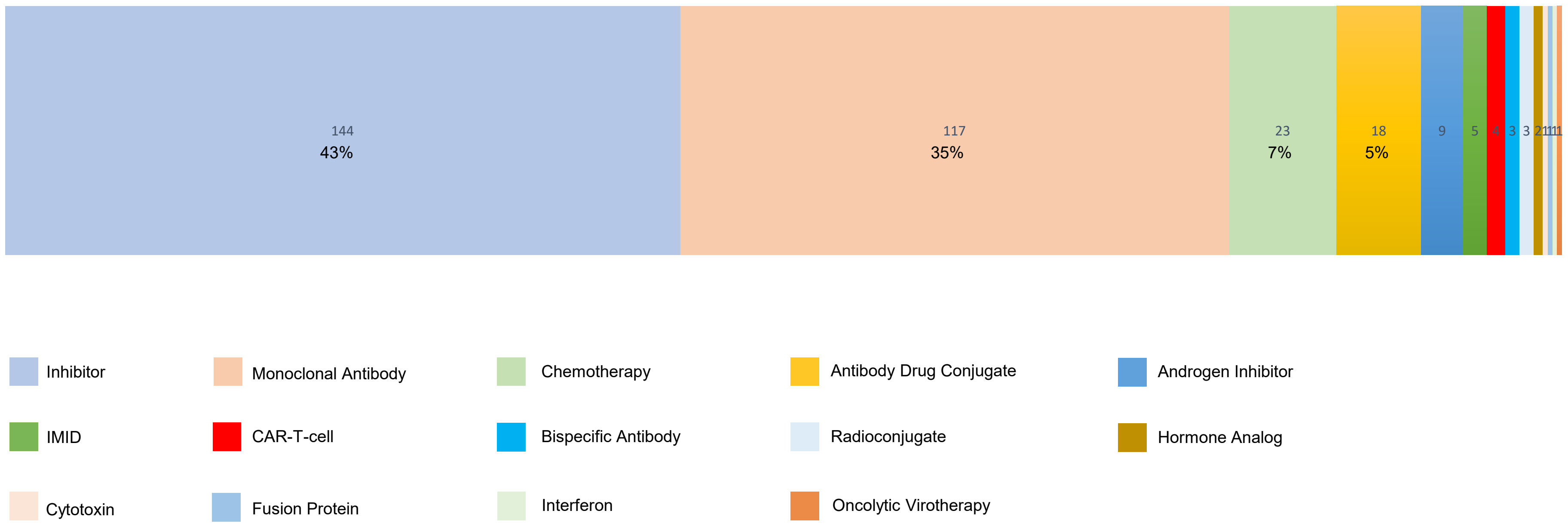

© 2021 Olivier T et al. *JAMA Network Open.*
